# Rab41 Is a Novel Regulator of Golgi Apparatus Organization That Is Needed for ER-To-Golgi Trafficking and Cell Growth

**DOI:** 10.1371/journal.pone.0071886

**Published:** 2013-08-06

**Authors:** Shijie Liu, Lauren Hunt, Brian Storrie

**Affiliations:** Department of Physiology and Biophysics, University of Arkansas for Medical Sciences, Little Rock, Arkansas, United States of America; Iowa State University, United States of America

## Abstract

**Background:**

The 60^+^ members of the mammalian Rab protein family group into subfamilies postulated to share common functionality. The Rab VI subfamily contains 5 Rab proteins, Rab6a/a’, Rab6b, Rab6c and Rab41. High-level knockdown of Rab6a/a’ has little effect on the tightly organized Golgi ribbon in HeLa cells as seen by fluorescence microscopy. In striking contrast, we found Rab41 was strongly required for normal Golgi ribbon organization.

**Methods/Results:**

Treatment of HeLa cells with Rab41 siRNAs scattered the Golgi ribbon into clustered, punctate Golgi elements. Overexpression of GDP-locked Rab41, but not wild type or GTP-locked Rab41, produced a similar Golgi phenotype. By electron microscopy, Rab41 depletion produced short, isolated Golgi stacks. Golgi-associated vesicles accumulated. At low expression levels, wild type and GTP-locked Rab41 showed little concentration in the Golgi region, but puncta were observed and most were in ruffled regions at the cell periphery. There was 25% co-localization of GTP-locked Rab41 with the ER marker, Sec61p. GDP-locked Rab41, as expected, displayed an entirely diffuse cytoplasmic distribution. Depletion of Rab41 or overexpression of GDP-locked Rab41 partially inhibited ER-to-Golgi transport of VSV-G protein. However, Rab41 knockdown had little, if any, effect on endosome-to-Golgi transport of SLTB. Additionally, after a 2-day delay, treatment with Rab41 siRNA inhibited cell growth, while overexpression of GDP-locked Rab41, but not wild type or GTP-locked Rab41, produced a rapid, progressive cell loss. In double knockdown experiments with Rab6, the Golgi ribbon was fragmented, a result consistent with Rab41 and Rab6 acting in parallel.

**Conclusion:**

We provide the first evidence for distinctive Rab41 effects on Golgi organization, ER-to-Golgi trafficking and cell growth. When combined with the evidence that Rab6a/a’ and Rab6b have diverse roles in Golgi function, while Rab6c regulates mitotic function, our data indicate that Rab VI subfamily members, although related by homology and structure, share limited functional conservation.

## Introduction

In most mammalian cell types, the Golgi apparatus (also known as the Golgi complex) exists as a juxtanuclear ribbon structure. This organized structure is generated by the interconnection of Golgi stacks consisting of a series of flattened, membrane-bound discs termed cisternae (for reviews, see 1,2). This highly organized Golgi structure is essential to normal protein glycosylation and sorting within the secretory pathway (for reviews, see 3,4). The Golgi apparatus occupies a central role in the secretory pathway and the anterograde and retrograde membrane trafficking pathways that converge at the Golgi apparatus (for reviews, see 5–7). Rab proteins, the largest family of small Ras-like GTPases, are associated with almost all steps of vesicle transport including those of the Golgi apparatus (for review, see 8). Among the 60 or more members of Rab protein family in mammalian cells, several of them including Rab6, Rab33b, Rabs1 and 2, Rab18 and Rab43 have been implicated in Golgi organization and trafficking (for review, see 9).

Rab6 is the most abundant Golgi-associated Rab protein in mammalian cells. Its four isoforms including Rab6a, Rab6a’, Rab6b and Rab6c, together with Rab41 constitute on the basis of homology the Rab VI subfamily [10]. These 5 proteins also group closely together on the basis of protein folding and surface charge exposure [11]. Rab6a and a’ are generated by alternate splicing of the primordial Rab6a/a’ gene on human chromosome 11 and differ in only three amino acid residues [12]. Rab6a and a’ are ubiquitously expressed in equal amounts, localized to the trans-Golgi cisternae and TGN membranes and have canonical GTP-binding domains [13–15]. They exhibit sufficiently similar biochemical and genetic properties that they are often collectively referred to as Rab6 [12]. Rab6b is coded by a gene located on chromosome 3. The identity between Rab6b and Rab6a is 91% and the protein is localized to the Golgi apparatus, ER and ER Golgi intermediate compartment (ERGIC). Unlike Rab6a/a’, Rab6b is preferentially expressed in brain. Rab6b also has canonical GTP-binding domains, although the GTP-binding activity of Rab6b is lower than that of Rab6a [16]. The identity between Rab6c and Rab6a’ is 75%; the lower homology is chiefly due to a 46-amino-acid extension at the COOH terminus of Rab6c. Rab6c is expressed in brain, testis, prostate and breast. GFP labeled Rab6c is predominantly associated with the centrosome, and unlike most other Rab proteins, it is not prenylated. In addition, Rab6c has a non-canonical GTP-binding domain, and its GTP-binding activity is greatly reduced [17]. The final Rab protein of this subfamily, Rab41, was proposed to be a Rab6-like protein due to its close homology and similar electrostatic potential [10,11]. However, experimentally this hypothesis is untested; the function and localization of Rab41 remain unknown.

Rab6 is the most extensively studied member of the subfamily. It is important to both anterograde and retrograde membrane trafficking within the juxtanuclear Golgi region of the cell [18–20]. Yet high depletion of Rab6 or overexpression of GDP-locked Rab6 in HeLa cells has little effect on Golgi ribbon structure as assayed by common fluorescence microscopy approaches [15,21] while, in mouse macrophages, the Golgi ribbon becomes somewhat more compact and in multinucleate cells may fragment [22]. These results suggest that Rab6 expression is not tightly linked to normal Golgi ribbon organization. However, in epistasis experiments, co-depletion of Rab6 inhibited Golgi ribbon disruption induced by retrograde tether protein Zeste White 10 (ZW10)/RINT-1 or conserved oligomeric Golgi (COG) knockdown, suggesting that Rab6 does have a role in the maintenance of Golgi organization [15]. Recently, Storrie et al [23] found by electron tomography that if Rab6 alone was depleted, Golgi cisternal number and cisternal continuity were increased, and both COPI- and clathrin-coated vesicles and coated membrane fission/fusion figures accumulated. In addition, Rab6 may well have important non-trafficking roles. Rab6a’ has been reported to have a role in the inactivation of the Mad2-spindle checkpoint in mitosis [24]. In comparison, the functional role of Golgi-associated Rab6b is little known beyond its implication in the complex trafficking pathways of neuronal cells [16]. Rab6c appears not to follow the overall postulate that members of a Rab subfamily are functionally related [10,11]. It is associated with centrosome duplication and cell cycle progression. Its involvement in apparent non-membrane trafficking process may well follow from its lack of prenylation and membrane association [17].

Here, we tested whether the least studied member of the Rab VI subfamily, Rab41, has a significant role in membrane trafficking and, in particular, in Golgi organization. In contrast to Rab6, we found by light microscopy and electron microscopy that Rab41 was strongly required for normal Golgi ribbon organization. By fluorescence microscopy, we found myc-tagged Rab41 in HeLa cells had little, if any, Golgi-association. Rather both wild type and GTP-locked Rab41 displayed diffuse fluorescence with occasional punctate localization in ruffled regions at the cell periphery. There was 25% co-localization of GTP-locked Rab41 with Sec61p, an ER marker. GDP-locked Rab41, as expected, showed an entirely diffuse cytoplasmic pattern. As a test of whether the effects on Golgi ribbon organization were a consequence of altered membrane trafficking, we tested the effect of Rab41 depletion or overexpression of GDP-locked Rab41 on VSV-G protein transport and found that much of that inhibition could be ascribed to an effect on ER-to-Golgi trafficking. Rab41 knockdown had little, if any, effect on SLTB transport from endosome to the Golgi apparatus. After a delay, treatment with Rab41 siRNA inhibited cell multiplication, while overexpression of GDP-locked Rab41, but not wild type or GTP-locked Rab41, produced a rapid, progressive cell loss. Together, these observations suggest that Rab41 is an essential protein. In double knockdown experiments with Rab6, the Golgi ribbon was fragmented, a result consistent with Rab41 and Rab6 acting in parallel. In conclusion, we provide the first functional data on Rab41 and show that, unlike Rab6a/a’, Rab41 strongly supports the maintenance of Golgi ribbon structure and ER-to-Golgi trafficking. These experiments provide further evidence that the extent of functional conservation within even a small Rab subfamily such as the Rab VI subfamily is limited.

## Results

**Figure 1:**
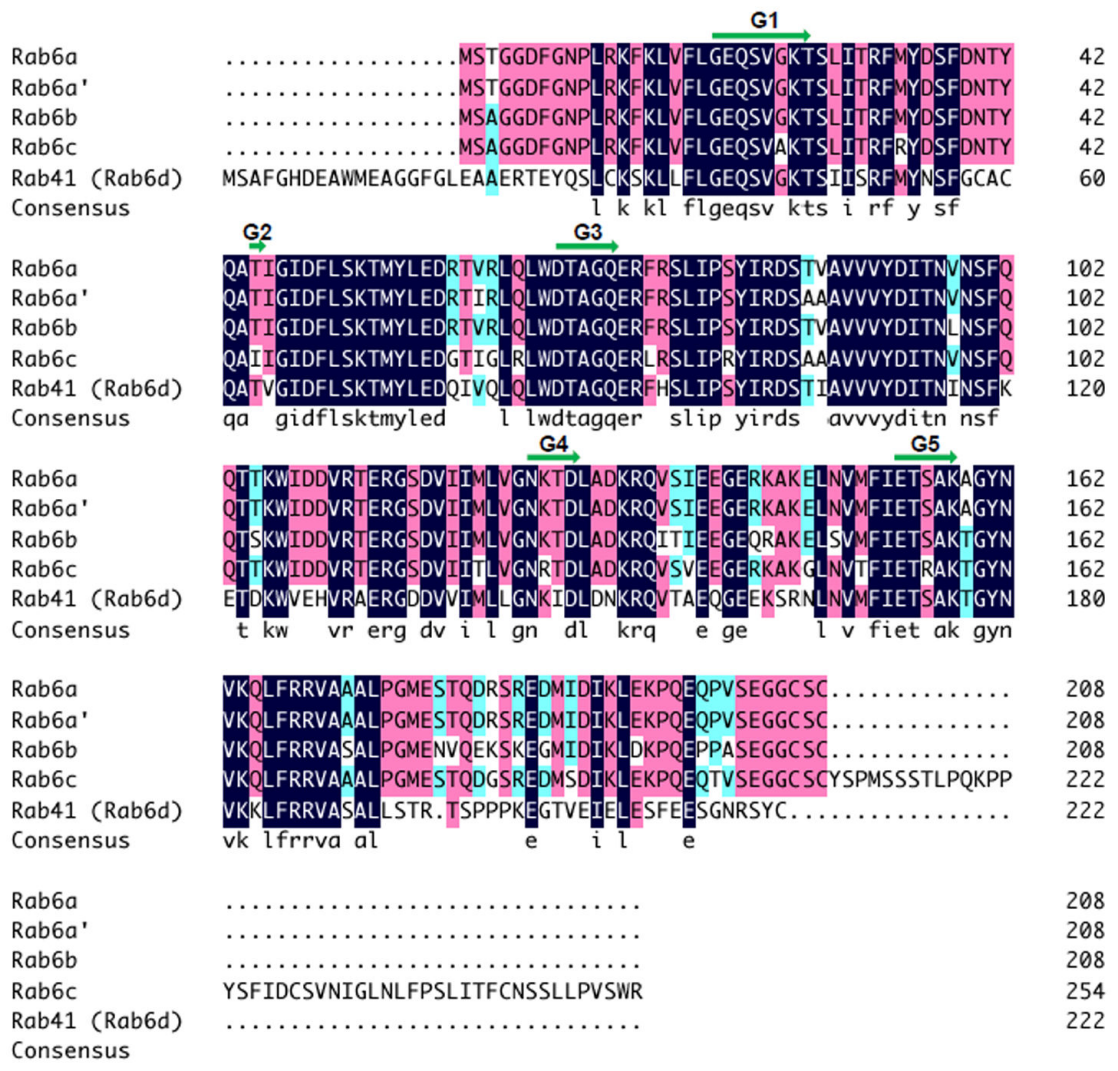
Alignment of amino acid sequences of five members of Rab VI subfamily. The multiple sequence alignment was performed with DNAMAN sequence analysis software. Consensus sequence among all five sequences is shown. The green arrows indicate GTP-binding regions (G1-G5) of Rab proteins.

Rab41, Rab6a, Rab6a’, Rab6b and Rab6c group together on the basis of homology in the Rab VI subfamily [10]. Similarly, if three-dimensional electrostatic and hydrophobic molecular interactions are considered, the proteins co-group [11]. In the multiple sequence alignment to determine which portions of Rab41 are shared with other members of the Rab VI subfamily, we found that amino acid segment 29 to 192, the central core sequence of the protein, had the greatest identity with other subfamily members, almost 80%. Overall, as shown in Figure 1, Rab41 shared a higher amino acid identity, ~64%, with Rab6b (64.2%) or Rab6a/a’ (63.7%/62.7%) than with Rab6c (58.8%). In sum, Rab41 showed greatest similarity with subfamily members that localize to the Golgi apparatus, intermediate compartment or ER. Based on this analysis, we reasoned that Rab41, a protein that could well be termed Rab6d, might associate with the Golgi apparatus and regulate its organization in a similar manner to the well-known Rab6 example.

### Both depletion and overexpression experiments indicate that Rab41 is actively required for Golgi ribbon organization

Two different approaches including depletion of Rab41 by Rab41 siRNAs and overexpression of GDP-locked Rab41 were used to test the function of Rab41 in Golgi organization. These led to the unexpected conclusion that, unlike Rab6, Rab41 is actively required for the maintenance of Golgi ribbon structure.

**Figure 2:**
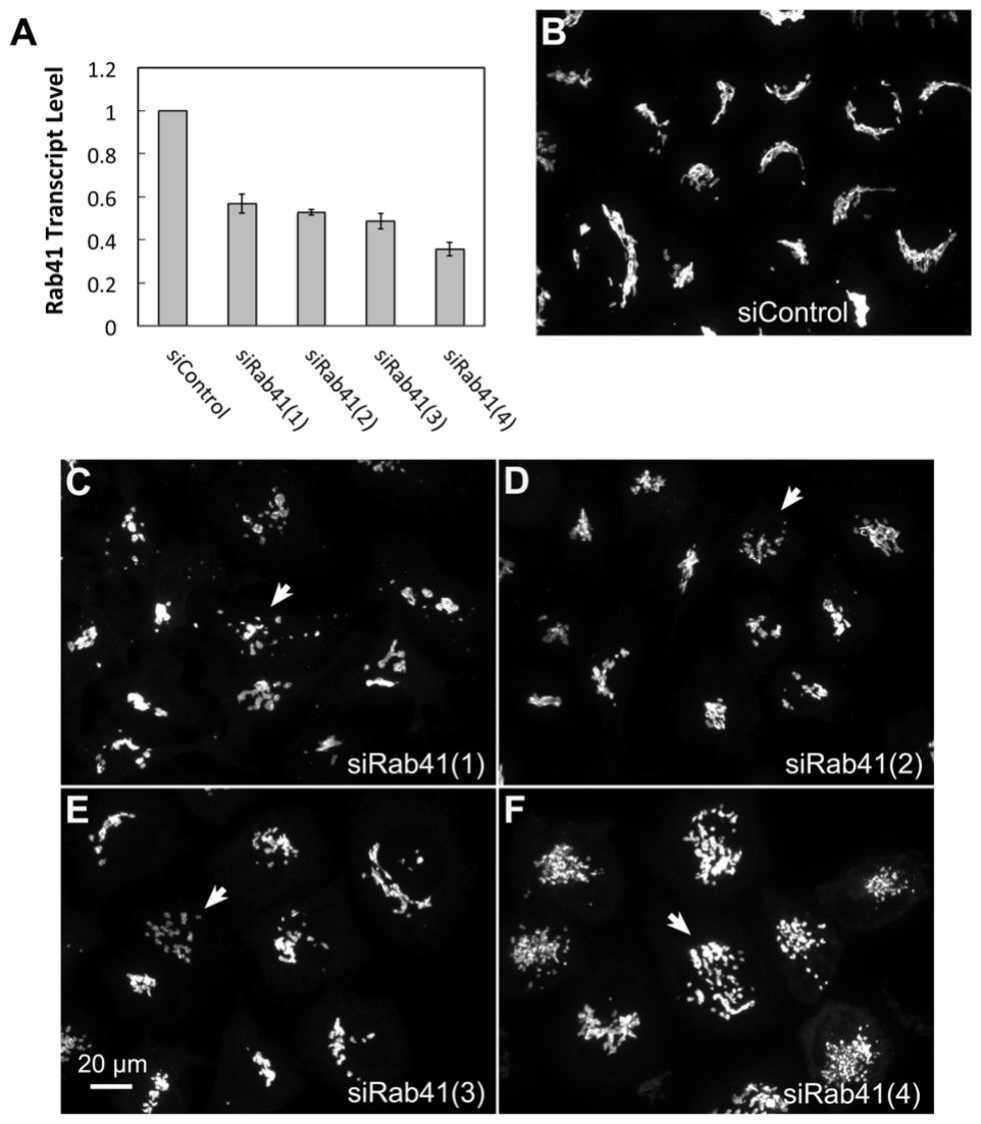
The effect of four Rab41 directed siRNAs on Rab41 mRNA level and Golgi ribbon organization. HeLa cells stably expressing GalNAcT2-GFP were transfected with either control or Rab41 siRNA at a concentration of 200 nM. HeLa cells were fixed or collected 96 h post initial transfection. Total RNA was isolated and transcribed to cDNA. Real-time PCR showed that, in cells treated by siRab41(1), siRab41(2) or siRab41(3), Rab41 transcript level was decreased ~40-50% relative to GADPH (as control), while in cells treated by siRab41(4), Rab41 transcript level was decreased >60%. Error bars represent the mean ± SEM of three replicates (A). In contrast with control (B), siRab41(1) caused mild level of Golgi disruption (C), siRab41(2) and siRab41(3) caused medium level of Golgi disruption (D and E), while siRab41(4) was the most effective and caused the highest level of Golgi disruption (F). Arrow in each image shows an example of fragmented Golgi apparatus produced by the corresponding Rab41 siRNA. SL and LH performed the same experiment independently and the results were the same.

We found that treatment of HeLa cells with siRNAs directed against Rab41 caused fragmentation of the Golgi ribbon into a clustered punctate Golgi distribution. The effects of four siRNAs corresponding to nucleotides 319-335 [siRab41(1)], 285-303 [siRab41(2)], 193-211 [siRab41(3)] and 360-378 [siRab41(4)] within the coding sequence of human Rab41 mRNA on both Rab41 transcript level and Golgi ribbon organization were determined. As shown in Figure 2B, the normal Golgi ribbon, indicated by the distribution of the stably expressed Golgi enzyme GalNAcT2-GFP, was juxtanuclear and compact in cells transfected with control siRNA. When cells were incubated with siRab41(1), the Rab41 transcript level was only decreased ~40% (Figure 2A), and only a mild level of Golgi disruption was observed (Figure 2C, arrow). At a higher decrease of Rab41 mRNA levels (~50%) in siRab41(2) and siRab41(3) treated cells (Figure 2A), the Golgi apparatus displayed a medium level of disruption (Figure 2D and 2E, arrows). The 4th siRNA, siRab41(4), caused >60% decrease of Rab41 transcript level (Figure 2A), and cells showed the highest level of Golgi disruption (Figure 2F, arrow). The above data suggests that, of these four siRNAs directed against Rab41, siRab41(4) treatment causes the greatest knockdown and the highest level of Golgi disruption. Therefore, siRab41(4) was used in all subsequent experiments.

**Figure 3:**
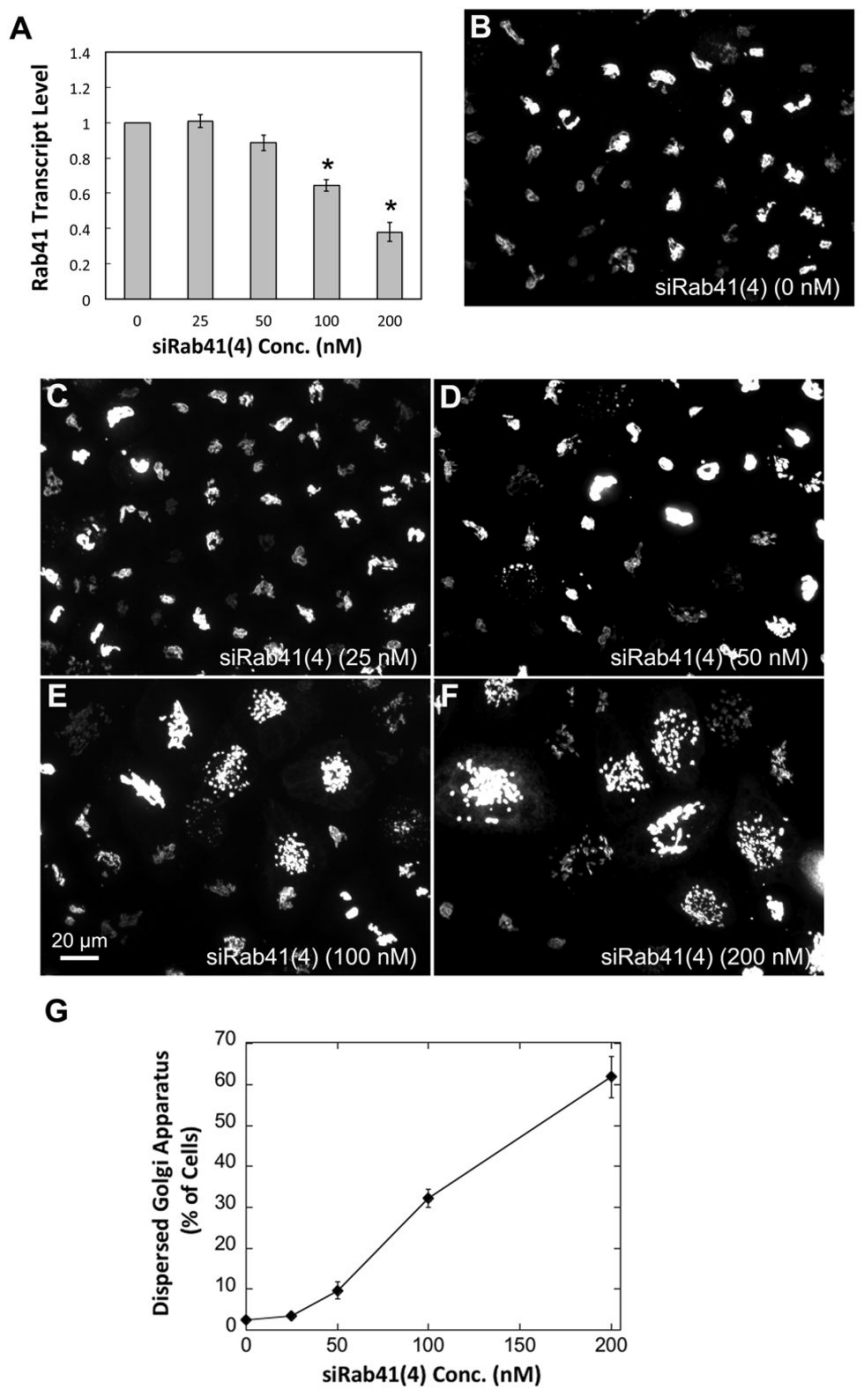
The knockdown efficacy of a range of concentrations of siRab41(4). HeLa cells stably expressing GalNAcT2-GFP were transfected with siRab41(4) at concentration of 0, 25, 50, 100 or 200 nM. HeLa cells were fixed or collected 96 h post initial transfection. Total RNA was isolated and transcribed to cDNA. Real-time PCR showed that, in cells treated by 100 nM and 200 nM siRab41(4), Rab41 transcript level was decreased ~30 and 60% relative to GADPH (as control), respectively (A, asterisks). Error bars in (A) represent the mean ± SEM of three replicates. Most of the cells transfected with 0, 25 or 50 nM siRab41(4) displayed normal Golgi ribbon structure (B–D). However, if the concentration of siRab41(4) was increased to 100 nM or 200 nM, fragmented Golgi apparatus was observed in many more cells (E and F). (G) Quantitatively, >30% cells treated with 100 nM siRab41(4) had clustered punctate Golgi distribution, and >60% cells transfected with 200 nM siRab41(4) displayed disrupted Golgi apparatus. Error bars represent the mean ± SEM of three replicates. ~100 cells were assayed for each condition and individual replicate.

We next tested the knockdown efficacy of siRab41(4) over a range of concentrations. As shown in Figure 3B, control cells exhibited a relatively compact Golgi ribbon as indicated by the distribution of the stably expressed, tagged Golgi protein GalNAcT2-GFP. As the concentration of siRab41(4) was increased from 25 or 50 nM to 100 and 200 nM, the transcript levels of Rab41 were significantly depressed. No obvious decrease in relative mRNA level for Rab41 was observed in cells treated with 25 or 50 nM siRab41(4) (Figure 3A). Correspondingly, Golgi ribbon organization appeared normal (Figure 3C and 3D). At higher siRNA concentrations, 100 or 200 nM, ~30% or 60% reduction, respectively, in transcript levels were determined (Figure 3A, asterisks). At these mRNA levels, many more cells displayed the clustered punctate Golgi phenotype (Figure 3E and 3F). Quantitatively, >30% of cells transfected with 100 nM siRab41(4) displayed fragmented Golgi apparatus, while by 200 nM siRab41(4) transfection, >60% of cells had clustered punctate Golgi distribution (Figure 3G). At all siRNA concentrations, an even greater extent of phenotype penetrance was observed if Lipofectamine 2000 was substituted for Oligofectamine as the transfection reagent (data not shown).

**Figure 4:**
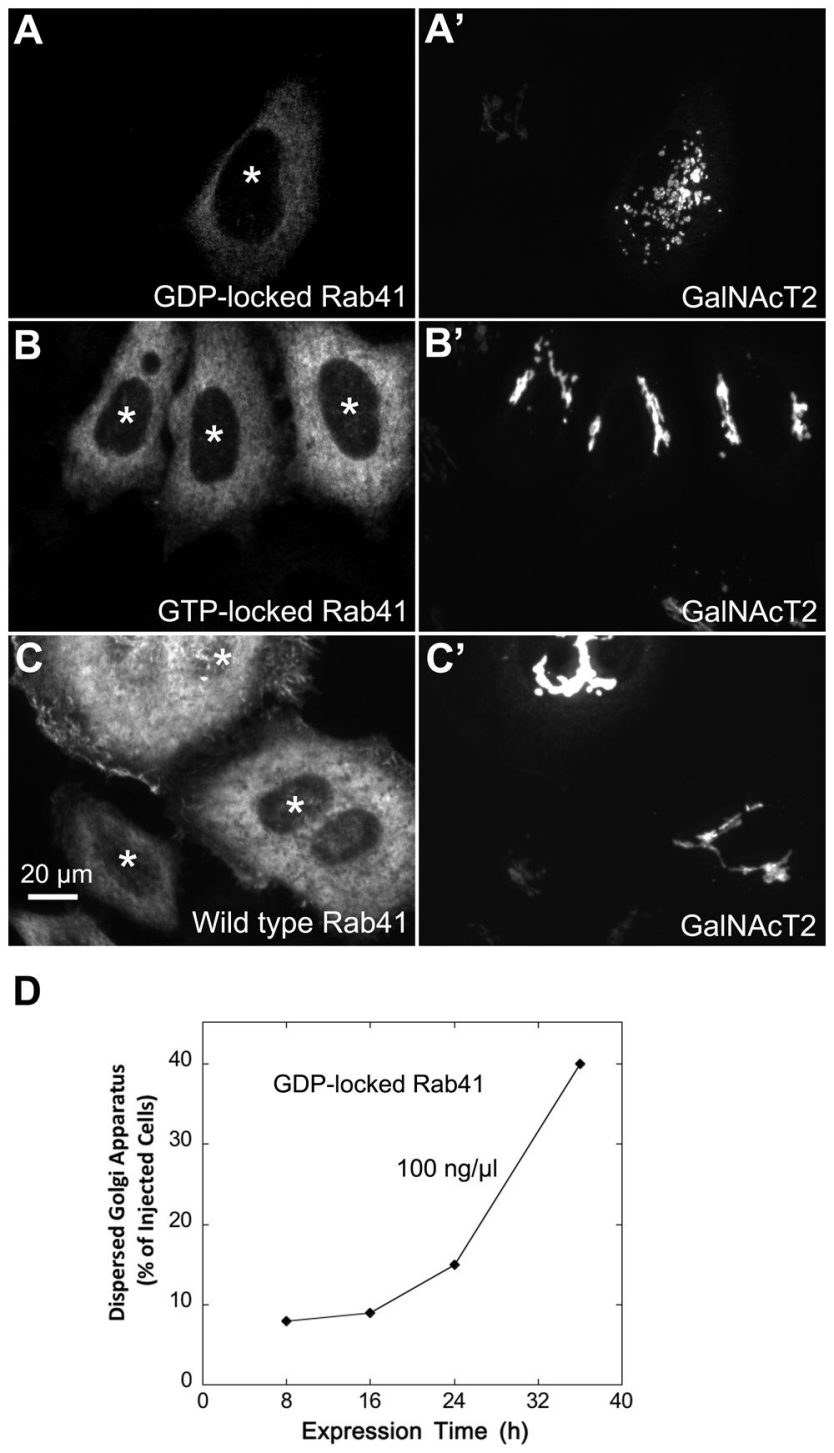
The effect of overexpression of wild type, GTP-locked and GDP-locked Rab41 on Golgi ribbon structure. HeLa cells stably expressing GalNAcT2-GFP were microinjected with pCMUIV plasmids encoding myc-tagged wild type, GTP-locked and GDP-locked Rab41, respectively. The stock plasmid concentration was 100 ng/µl. After a 36-h expression period, cells were fixed and stained with anti-myc antibody (A–C). Golgi structure was displayed by expression of Golgi-associated protein, GalNAcT2-GFP (A’–C’). In cells overexpressing GDP-locked Rab41, the Golgi apparatus was fragmented (A and A’), whereas the compact Golgi ribbon was observed in cells injected by either GTP-locked (B and B’) or wild type Rab41 (C and C’). Asterisks mark injected cells. (D) Quantitative analysis of cells overexpressing GDP-locked Rab41 displayed fragmented Golgi apparatus at 100 ng/µl plasmid concentration and ranged expression time from 8 to 36 h. ~50 injected cells were assayed for each time point.

To further validate the involvement of Rab41 in maintenance of Golgi ribbon structure, we tested the effects of overexpression of myc-tagged wild type, GTP-locked and GDP-locked Rab41 on Golgi ribbon organization. For GTP-locked Rab41, the glutamine residue at position 90 was replaced by leucine and for GDP-locked Rab41, the threonine residue at position 45 was replaced by asparagine [25]. Plasmids encoding myc-tagged versions of each of these proteins were introduced into HeLa cells stably expressing the Golgi enzyme GalNAcT2-GFP by microinjection. Tagged Rab41 expression was assayed by antibody staining. As shown in Figure 4, the overexpression of wild type or GTP-locked Rab41 had little, if any, effect on juxtanuclear distribution of Golgi apparatus, whereas overexpressed GDP-locked Rab41 induced Golgi ribbon fragmentation to a cluster of Golgi elements. To optimize the incidence of Golgi disruption, a range of plasmid concentrations and expression times were tested for GDP-locked Rab41. Optimal results were obtained at a plasmid concentration of 100 ng/µl; the frequency of injected cells that displayed fragmented Golgi apparatus increased progressively as the expression period increased from 8 to 36 h. At 36-h expression time, the Golgi apparatus was fragmented in ~40% of the injected cells overexpressing GDP-locked Rab41 (Figure 4D). Higher plasmid concentrations were prohibitively toxic (see Result section: “Rab41 is essential for HeLa cell multiplication”).

**Figure 5:**
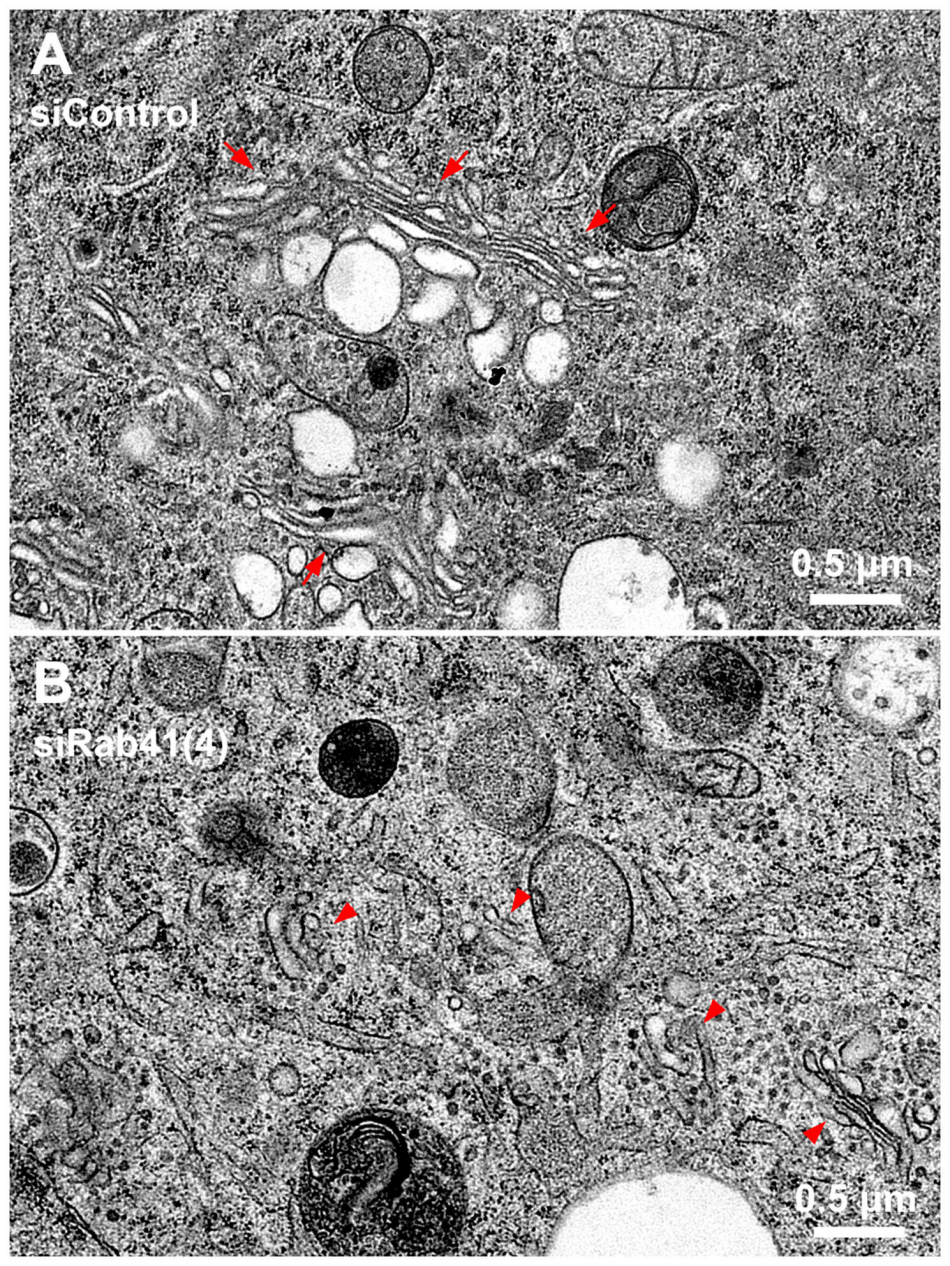
Electron microscopy demonstrates that Rab41 depletion causes dramatic Golgi fragmentation and accumulation of Golgi-associated vesicles. HeLa cells stably expressing GalNAcT2-GFP grown on sapphire discs were treated with either control siRNA or siRab41(4) duplexes at a concentration of 200 nM. Cells were high-pressure frozen followed by freeze-substitution 96 h post initial transfection. Thin sections (50 nm) were collected to test the Golgi phenotype. In cells transfected with control siRNA, Golgi stacks were close enough to form a long Golgi ribbon-like structure (A, arrows). However, in cells treated with siRab41(4), short, isolated Golgi stacks (B, arrowheads) rather than Golgi ribbon were observed, and Golgi-associated vesicles accumulated (B).

In sum, results from two different approaches, siRNA and mutant overexpression, indicate that Rab41 does have an important function in Golgi ribbon organization. However, and unexpectedly, the phenotype produced is quite different from that of Rab6 inactivation. Rab41 inactivation results in Golgi ribbon scattering. Rab6 inactivation, if anything, results in a more organized Golgi ribbon [15]. To test what might be true of individual Golgi cisternae and Golgi-associated vesicles, we used the decidedly higher resolution approach of electron microscopy. Control and Rab41 siRNA treated cells were cultured on sapphire discs and high-pressure frozen in situ followed by freeze-substitution. The Golgi apparatus in control cells consisted of closely spaced Golgi stacks composed of several flattened cisternae. Stacks were close enough to each other to form a larger ribbon-like structure (Figure 5A, arrows). The Golgi apparatus in Rab41 depleted cells exhibited a much different phenotype. In fact, no ribbon-like structures in Rab41 depleted cells were found, rather short, isolated Golgi stacks were observed (Figure 5B, arrowheads). Moreover, the frequency of Golgi-associated vesicles also appeared higher in Rab41 depleted cells (Figure 5). To quantify these results, electron micrographs from two separate experiments were analyzed across a minimum of 18 different Golgi stacks per experiment (Table 1). In control cells, the average number of stacks per Golgi rich area was 3.5 and the average maximum cisternae length was ~900 nm, whereas in Rab41 depleted cells, the number of stacks per Golgi rich area was increased, 5.4, and the maximal length of Golgi cisternae was significantly reduced, ~400 nm. Moreover, in contrast with control cells, the average number of Golgi-associated vesicles per stack in Rab41 knockdown cells was ~2-fold higher. In addition, a decrease was observed for the average number of cisternae per stack in Rab41 knockdown cells. Hence, results from electron microscopy further demonstrate the active role of Rab41 in maintenance of Golgi ribbon structure and individual Golgi cisternal stacks.

**Table 1:** Quantification of Golgi morphology in siControl and siRab41(4) transfected cells.

**Experiment 1**					
siControl Golgi					
Cell	Number of stacks per Golgi rich area	Average number of Golgi-associated vesicles per stack	Average maximum cisternae length (nm)	Average Golgi stack width (nm)	Average number of cisternae per stack
1	4	5	1022	240	4
2	3	10	1287	403	4
3	3	7	747	198	3
4	5	8	912	296	3
5	3	7	1084	325	4
Average ± SEM	3.6 ± 0.4	7.4 ± 0.8	1010.4 ± 89.8	292.4 ± 35.3	3.6 ± 0.2
siRab41(4) Golgi					
1	6	13	405	254	3
2	4	14	380	243	2
3	4	13	356	342	3
4	6	12	426	375	2
5	5	13	356	287	3
6	3	17	473	285	3.00
Average ± SEM	4.7 ± 0.5	13.7 ± 0.7	399.3 ± 18.5	297.7 ± 20.9	2.7 ± 0.2
**Experiment 2**					
siControl Golgi					
1	4	9	865	312	3
2	2	5	803	240	5
3	2	9	1078	334	4
4	4	8	777	301	4
5	3	7	841	321	4
6	5	9	770	310	3
Average ± SEM	3.3 ± 0.5	7.8 ± 0.7	855.7 ± 46.9	303.0 ± 13.4	3.8 ± 0.3
siRab41(4) Golgi					
1	5	18	500	314	3
2	5	9	384	290	2
3	8	15	501	255	3
4	6	11	399	309	3
5	4	15	403	223	2
6	9	11	464	298	2
7	7	12	485	322	3
8	5	14	452	286	2
Average ± SEM	6.1 ± 0.6	13.1 ± 1.0	448.5 ± 16.7	287.1 ± 11.7	2.5 ± 0.19
**Overall Average**					
**siControl Golgi**	**3.5**	**7.6**	**933.0**	**297.7**	**3.7**
**siRab41(4) Golgi**	**5.4**	**13.4**	**423.9**	**292.4**	**2.6**

### Rab41 is needed for rapid ER-to-Golgi trafficking and overall ER-to-plasma membrane trafficking

**Figure 6:**
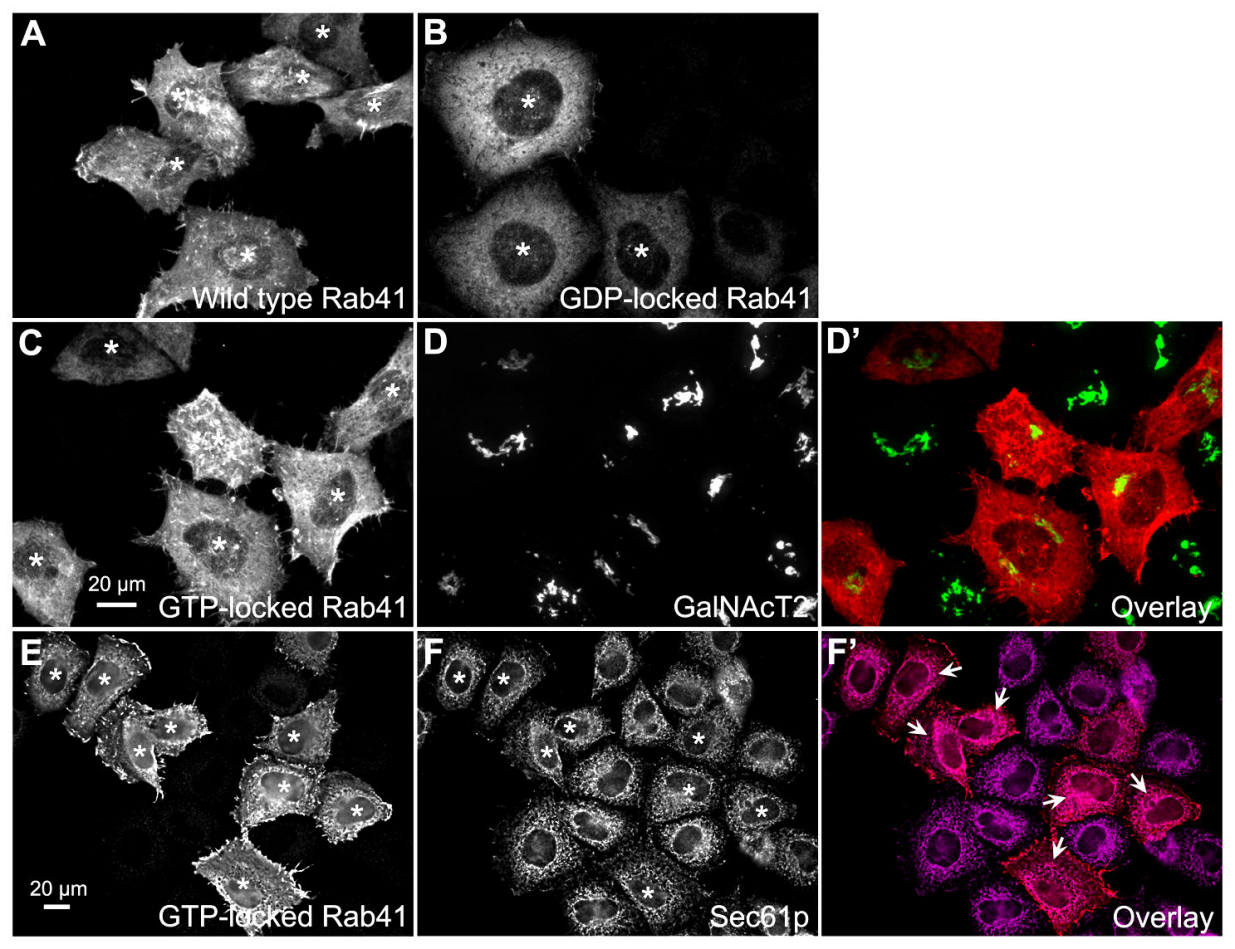
Distribution of wild type, GTP-locked and GDP-locked Rab41 in HeLa cells. HeLa cells stably expressing GalNAcT2-GFP were microinjected with pCMUIV plasmids encoding myc-tagged wild type, GTP-locked and GDP-locked Rab41, respectively. The stock plasmid concentration was 25 ng/µl. After a 1-h expression period, cells were fixed and then stained with anti-myc antibody. Wild type, GTP-locked and GDP-locked Rab41 were all observed to be distributed in cytoplasm rather than Golgi apparatus and nucleus. Moreover, wild type and GTP-locked Rab41 showed much more punctate localization, rather most puncta were concentrated in ruffled regions at the cell periphery (A and C). However, GDP-locked Rab41 displayed an entirely diffuse cytoplasm distribution (B). In the overlay image (D’), distribution of GTP-locked Rab41 (C) is shown in red, whereas the Golgi apparatus displayed by the expression of GalNAcT2-GFP (D) is shown in green. To test ER localization of GTP-locked Rab41, cells were double immunostained for myc-tagged Rab41 and Sec61p (ER marker). Immunostaining revealed limited overlap with any co-localization observed restricted to some areas around the cell nucleus (F’, arrows). In the overlay image (F’), distribution of GTP-locked Rab41 (E) and Sec61p (F) were shown in red and magenta, respectively. Images shown in A-D’ are single plane projections of confocal image stacks through the full cell depth, whereas images shown in E-F’ are single-plane deconvolved, wide field micrographs. Asterisks mark injected cells.

We next determined if myc-tagged Rab41 in HeLa cells was a Golgi-associated protein. The canonical RabVI family member, Rab6, is predominantly Golgi-associated [13–15]. We assessed the distribution of plasmid-encoded wild type, GTP-locked and GDP-locked Rab41 in HeLa cells using a low injected plasmid concentration (25 ng/µl) and a short expression time (1 h). The goal was to achieve localization at the lowest expression levels manageable. As shown in Figure 6, wild type, GTP-locked and GDP-locked Rab41 were all observed to be cytoplasmic with little concentration in the Golgi region. Note that Rab41 is excluded from the nucleus. Wild type and GTP-locked Rab41 had a much more punctate distribution than GDP-locked Rab41. Stained puncta were often concentrated in ruffled regions at the cell periphery (Figure 6A, 6C and 6E). GDP-locked Rab41, as expected, showed an entirely diffuse cytoplasmic pattern (Figure 6B). ER co-localization of GTP-locked Rab41 was tested against Sec61p as an ER marker. The immunostaining pattern of GTP-locked Rab41 was not overall very similar to that of endogenous Sec61p (Figure 6E-F’). However, some co-localization of the two was apparent around the cell nucleus (Figure 6F’, arrows) and, when quantified, 25% of the GTP-locked Rab41 co-localized with Sec61p. In sum, this result places a portion of Rab41 at a site potentially important to ER-to-Golgi transport.

**Figure 7:**
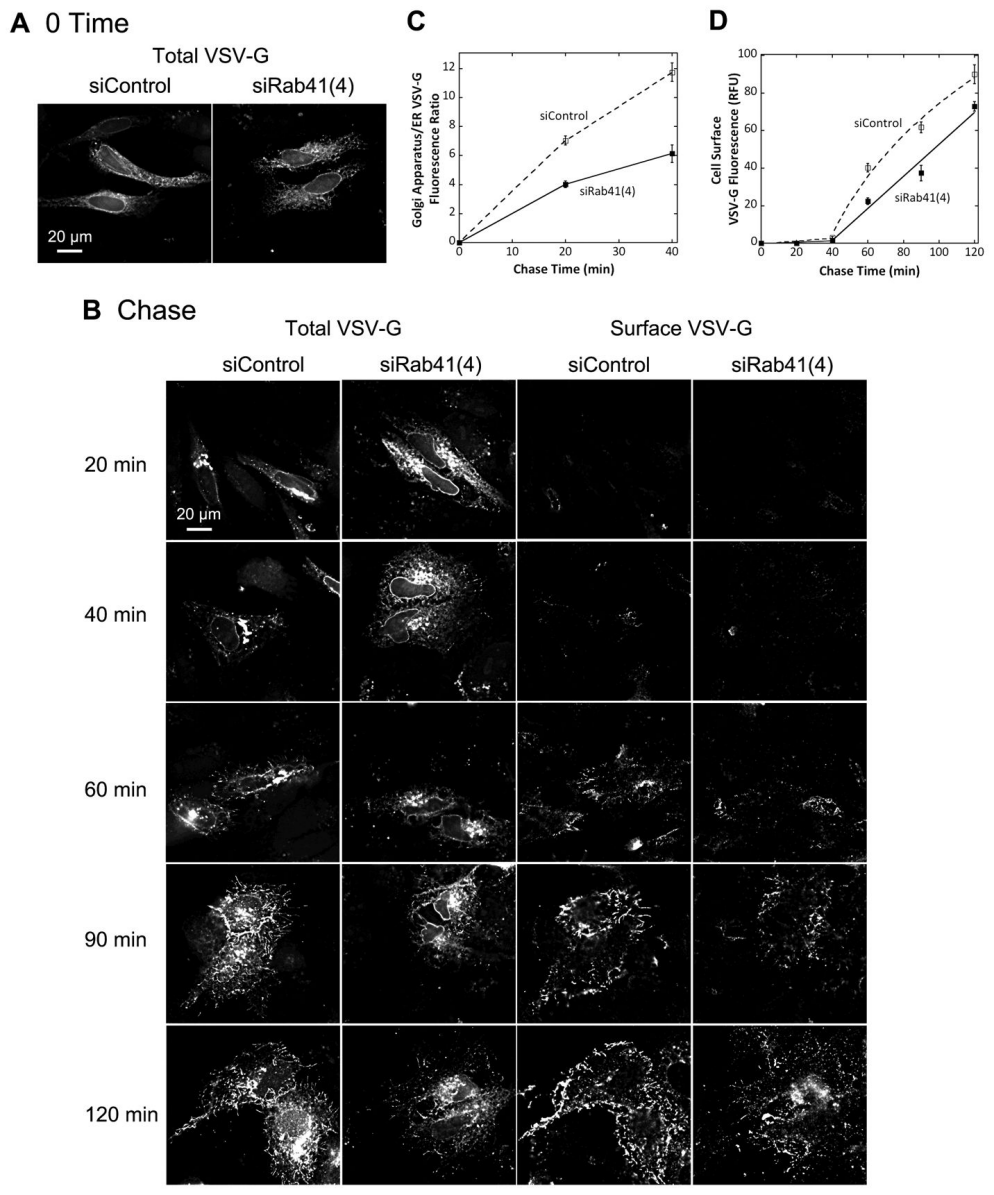
Depletion of Rab41 partially inhibited VSV-G transport from ER to the cell surface with a significant inhibition of ER to Golgi transport. Wild type HeLa cells were incubated with either siRab41(4) or non-targeting siRNA duplexes for 96 h and then transfected with plasmid encoding VSV-G-GFP. At the end of the 39.5° C incubation period, VSV-G was accumulated in the ER (A). Cells were then shifted to 32° C, permissive conditions for VSV-G transport, and incubated for various chase time in the presence of cycloheximide to prevent further protein synthesis (B). Cells were then fixed and cell surface stained for VSV-G, and visualized by wide field light microscopy. At the end of a 20-min chase or 40-min chase, Golgi accumulation of VSV-G was observed in both control and Rab41 knockdown cells. However, VSV-G retention in the ER of Rab41 depleted cells was decidedly higher than that of control cells (total VSV-G, left two columns in B, and C). Consistently, at later chase times, surface accumulation of VSV-G in Rab41 knockdown cells was quantitatively slower than that in control cells (surface VSV-G, right two columns in B, and D). All images shown or used for quantification were single-plane deconvolved. Error bars are the mean ± St Dev of three independent experiments. ~30 cells were assayed for each time point in the individual experiments.

To provide further functional characterization of Rab41, we turned our focus to the possible consequences of Rab41 knockdown on anterograde cargo trafficking. The transport of the model cargo protein, VSV-G, in control cells and Rab41 depleted cells was assessed using the tsO45 temperature sensitive mutant of VSV-G protein. The mutant protein accumulates in the ER at 39.5° C, and following a temperature shift to 32° C then traffics via the Golgi apparatus to the plasma membrane. At the end of the 39.5° C period, the GFP-tagged VSV-G mutant was distributed uniformly in the ER in both control and Rab41 knockdown cells (Figure 7A). Following a temperature shift to 32° C, the clearance of tsO45-G protein from the ER to Golgi apparatus appeared to be slower in Rab41 depleted cells than control cells (Figure 7B, 20 min and 40 min, left two columns). Quantitatively, the ratio of Golgi-associated VSV-G fluorescence to ER- associated VSV-G fluorescence was almost 2-fold higher in control cells than in Rab41 knockdown cells (Figure 7C). At later time points, we analyzed quantitatively only VSV-G accumulation at the cell surface using an antibody specific for a VSV-G ecto-epitope. Overall, tsO45-G at the cell surface was slower in Rab41 knockdown cells than control, ~18% less even at 120-min chase time (Figure 7B, right two columns, and 7D). These results indicate that depletion of Rab41 caused an inhibition of ER-to-cell surface cargo transport with a significant fraction of that inhibition being due to slower ER-to-Golgi transport. Whether this outcome is a primary effect of Rab41 depletion or a secondary effect of Golgi disorganization in the Rab-depleted cell is for now an open question.

**Figure 8:**
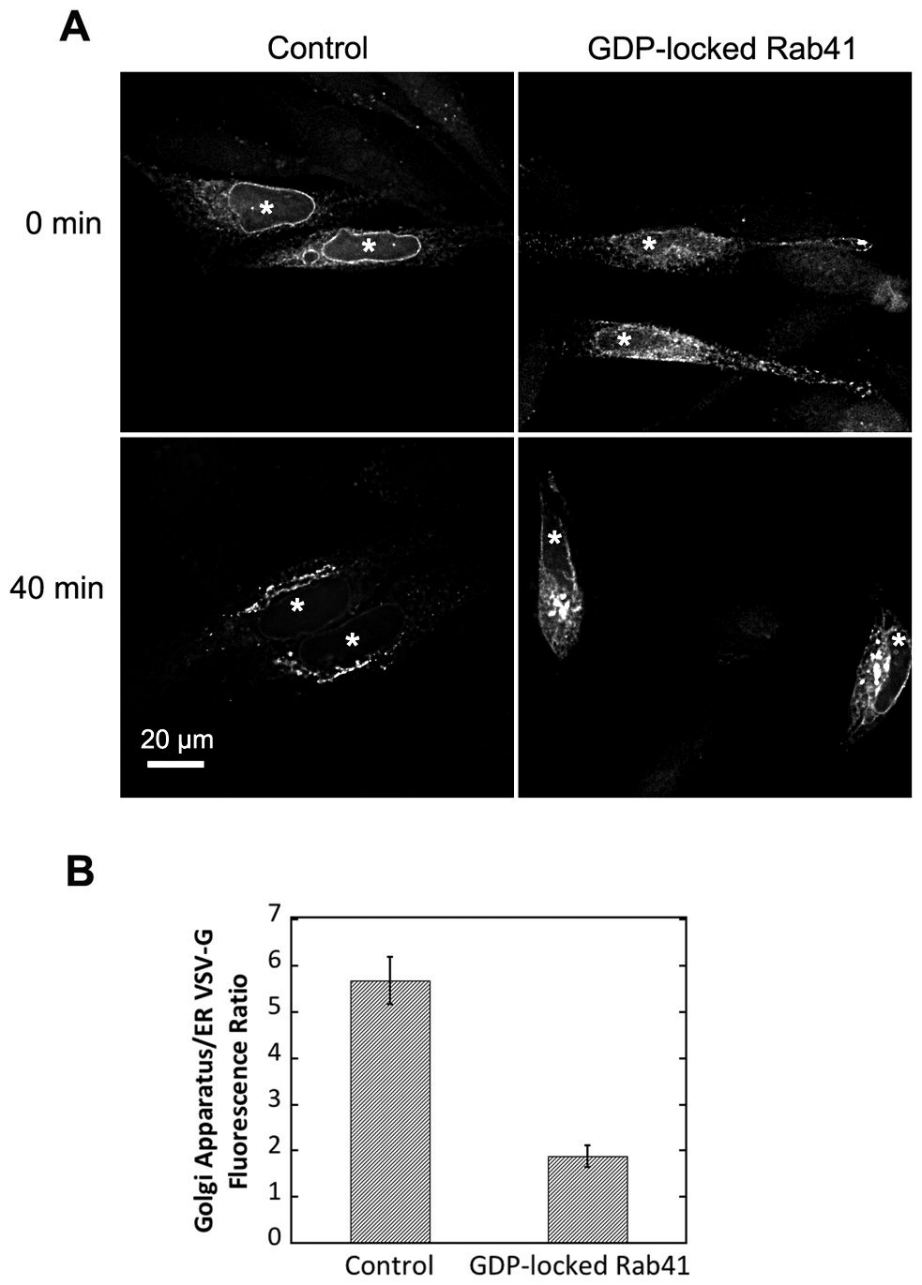
Overexpression of GDP-locked Rab41 partially inhibited VSV-G transport from ER to Golgi apparatus. Wild type HeLa cells were microinjected with either 25 ng/µl plasmid encoding the tsO45 mutant of VSV-G-GFP (Control) or a mixture of 100 ng/µl myc-tagged GDP-locked Rab41 encoding plasmid and 25 ng/µl tsO45 mutant of VSV-G-GFP encoding plasmid. After 24-h incubation at 39.5° C, VSV-G was accumulated in the ER (A, upper panel). Cells were then shifted to 32° C, permissive conditions for VSV-G transport, and incubated for 40 min in the presence of cycloheximide to prevent further protein synthesis (A, lower panel). Cells were then fixed and visualized by wide field light microscopy. At the end of a 40-min chase, Golgi accumulation of VSV-G was observed in both control and GDP-locked Rab41 overexpressing cells. However, VSV-G retention in the ER for GDP-locked Rab41 overexpressing cells was much higher than that in control cells (A, lower panel, and B). Successful co-injection was confirmed by antibody staining. All images shown or used for quantification were single-plane deconvolved. Error bars represent the mean ± SEM of ~20 injected cells. Asterisks mark injected cells.

We also tested the effect of overexpression of GDP-locked Rab41 on VSV-G transport from ER to Golgi apparatus. Plasmids encoding myc-tagged GDP-locked Rab41 and/or the tsO45 mutant of VSV-G-GFP were co-injected into wild type HeLa cells. Tagged Rab41 expression was assayed by antibody staining (data not shown). Cells injected only with plasmid encoding the tsO45 mutant of VSV-G-GFP were used as control. Initially, the GFP-tagged VSV-G mutant was accumulated in the ER (Figure 8A, 0 min). After a 40-min chase at 32° C, transport of tsO45-G protein from ER to Golgi apparatus was significantly delayed in cells overexpressing GDP-locked Rab41 (Figure 8A, 40 min). Quantitatively, the ratio of Golgi-associated VSV-G fluorescence to ER- associated VSV-G fluorescence was almost 3.5-fold higher in control cells than in Rab41 knockdown cells (Figure 8B). This result provides additional evidence from an alternate approach for the involvement of Rab41 in ER-to-Golgi trafficking.

**Figure 9:**
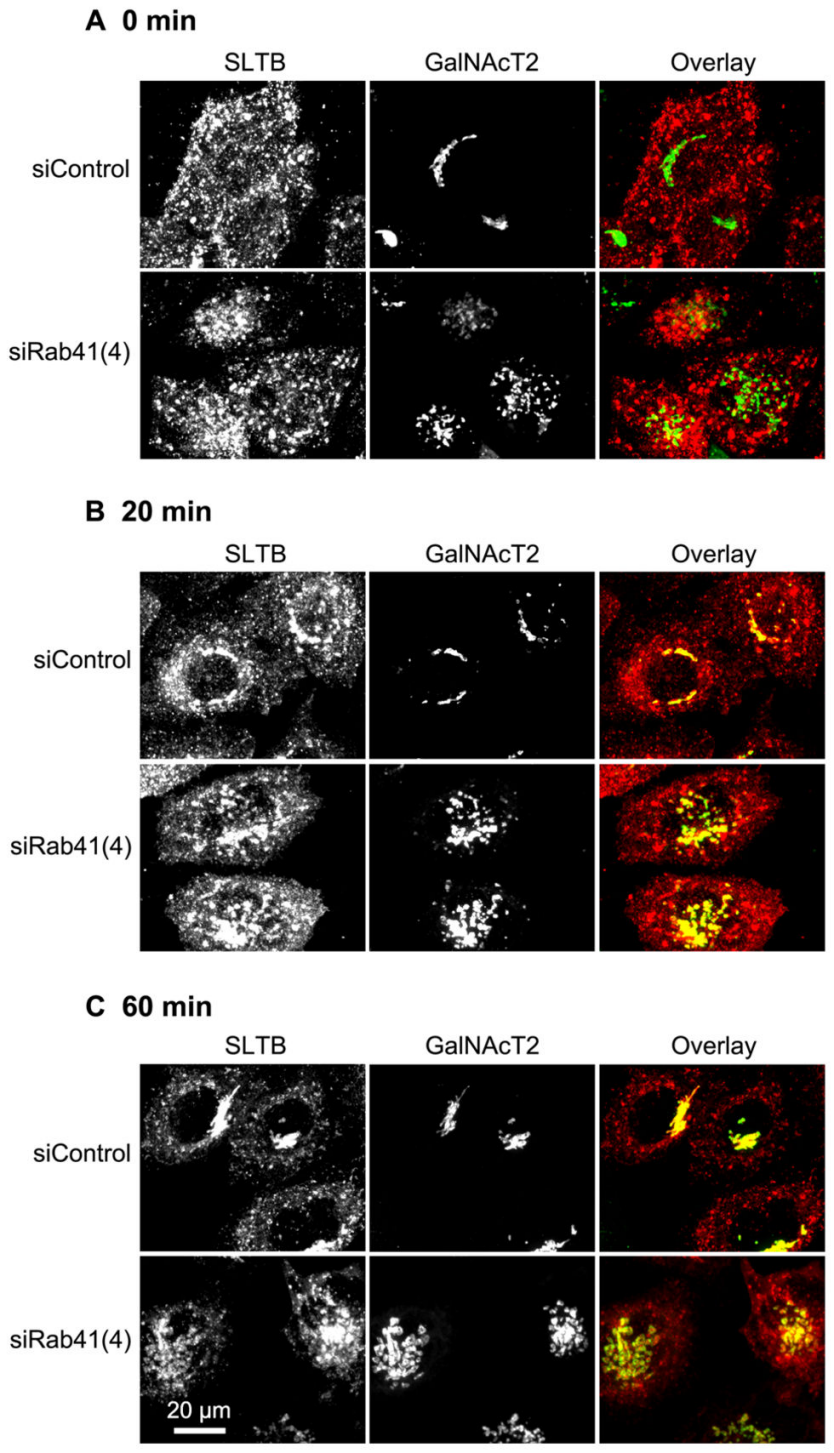
Rab41 knockdown has little effect on the transport of SLTB from endosome to Golgi apparatus. HeLa cells stably expressing GalNAcT2-GFP were transfected with either control siRNA or siRab41(4) duplexes at a concentration of 200 nM. 96 h post initial transfection, cells were incubated with Cy3-SLTB at 4° C for 30 min and then transferred to 19.5° C, and incubated for 1 h to accumulate Cy3-SLTB in endosomes (A). Cells were then shifted to 37° C, permissive temperature for Cy3-SLTB transport from endosomes to Golgi apparatus, and incubated for 20 or 60 min before being fixed. At the end of 20-min (B) or 60-min chase (C), Cy3-SLTB accumulated in Golgi apparatus in both control and Rab41 knockdown cells, indicating that Rab41 knockdown has little effect on the transport of SLTB from endosome to Golgi apparatus. In the overlay images, distribution of Cy3-SLTB was shown in red, whereas the Golgi apparatus displayed by the expression of GalNAcT2-GFP was shown in green.

To investigate whether Rab41 is required for endosome-associated membrane trafficking pathways, we used Shiga-like toxin B fragment (SLTB) as a tracer for endosome-to-Golgi trafficking. HeLa cells stably expressing GalNAcT2-GFP were treated with either control siRNA or siRab41(4). 4 days post transfection, cells were incubated with Cy3-SLTB at 4° C to allow binding of Cy3-SLTB to the glycolipid Gb3 at the cell surface. Cells were then transferred to 19.5° C, a permissive temperature for accumulation of Cy3-SLTB in endosomes (Figure 9A). Following a shift to 37° C, Cy3-SLTB was transported from endosomes to the Golgi apparatus. After a 20-min chase in both control and Rab41 knockdown cells, Cy3-SLTB fluorescence was observed in both endosomes and Golgi apparatus with no obvious difference in distribution between the two cases (Figure 9B). With further incubation at 37° C, 60-min chase, little to no difference was observed. At the end of a 60-min chase, in both control and Rab41 knockdown cells, Cy3-SLTB continued to be transported to the Golgi apparatus and endosome- associated Cy3-SLTB decreased to very low levels (Figure 9C). Based on these results, we conclude that Rab41 depletion had little effect on endosome-to-Golgi trafficking.

### Rab41 is essential for HeLa cell multiplication

**Figure 10:**
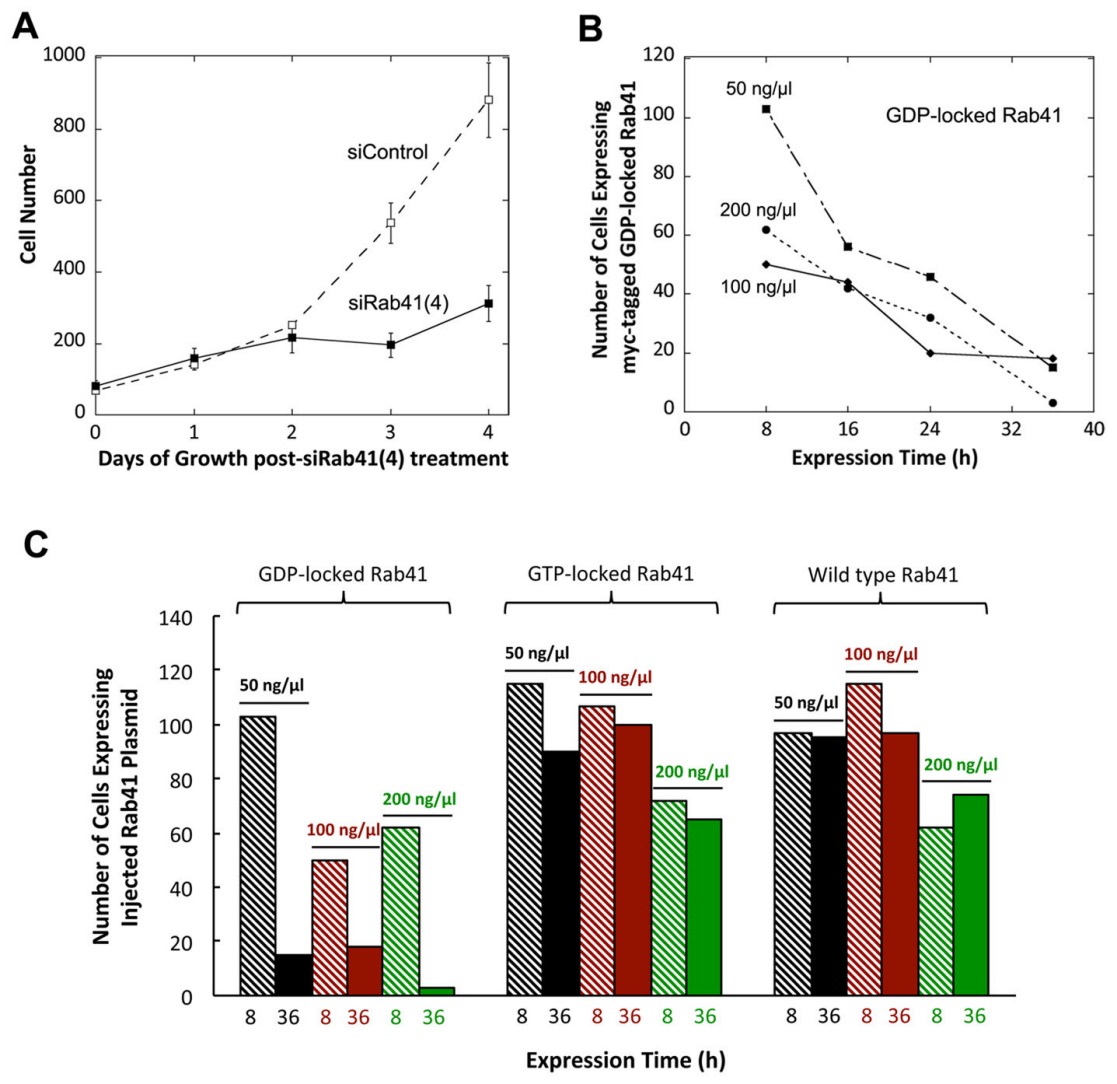
The effect of Rab41 knockdown or overexpression on HeLa cell growth. In knockdown experiments, ~70,000 HeLa cells stably expressing GalNAcT2-GFP were transfected with either control siRNA or siRab41(4) at a concentration of 200 nM. After a 2-day delay, the siRab41(4) treated cultures showed little further increase in cell number (A). As cell number shown on the Y-axis in A is the mean of the 6 image fields sample, the total number of cells scored at each time point is 6 times the Y axis number. In microinjection experiments, HeLa cells stably expressing GalNAcT2-GFP were microinjected with a range of concentrations (50, 100 and 200 ng/µl) of pCMUIV plasmids encoding myc-tagged wild type, GTP-locked or GDP-locked Rab41. After a period of expression at 37° C, cells were fixed and stained with anti-myc antibody. (B) Number of cells overexpressing GDP-locked Rab41 at various times following plasmid injection. A constant number of cells were injected in all cases. (C) Quantitative analysis of cell survival for HeLa cells microinjected with wild type, GTP-locked or GDP-locked Rab41 following 8-h or 36-h plasmid expression. Error bars represent the mean ± range of two independent experiments.

As an additional test of the cellular role of Rab41, we determined the effects of Rab41 depletion or overexpression on HeLa cell growth. In knockdown experiments, two cycles of transfection were performed using 200 nM control siRNA or siRab41(4). Cells were counted manually on each day post initial transfection. As shown in Figure 10A, the number of control cells approximately doubled in number each day. For siRab41(4) treated cells, a near doubling in cell number was observed initially, but by 2 days post initial transfection, growth of Rab41 depleted cells slowed considerably. At 3 or 4 days post initial transfection, the number of cells in siRab41(4) treated cultures was <50% of control. Hence, knockdown of Rab41 using siRab41(4) significantly inhibited cell growth. However, cell morphology was not affected (data not shown). In microinjection experiments, expression of wild type or mutant Rab41 in HeLa cells was tested at various plasmid concentrations and expression times. At each plasmid concentration used (50, 100 or 200 ng/µl), the number of cells overexpressing GDP-locked Rab41 decreased progressively as the expression time increased from 8 h to 16, 24 or 36 h. At 200 ng/µl plasmid concentration and 36-h expression time, few injected cells overexpressing GDP-locked Rab41 remained (Figure 10B and 10C). Importantly, at all plasmid concentrations, little to no decrease in the number of cells overexpressing wild type or GTP-locked Rab41 was observed at 8-h and 36-h expression period. Only at 200 ng/µl plasmid concentration was a mild decrease in the number of cells overexpressing wild type or GTP-locked Rab41 observed (Figure 10C). Based on these data, we conclude that Rab41 is not only needed for maintenance of Golgi ribbon structure and ER-to-Golgi trafficking, but is also crucial to HeLa cell growth and multiplication.

### The Golgi fragmentation induced by Rab41 knockdown is not suppressed by depletion of Rab6

**Figure 11:**
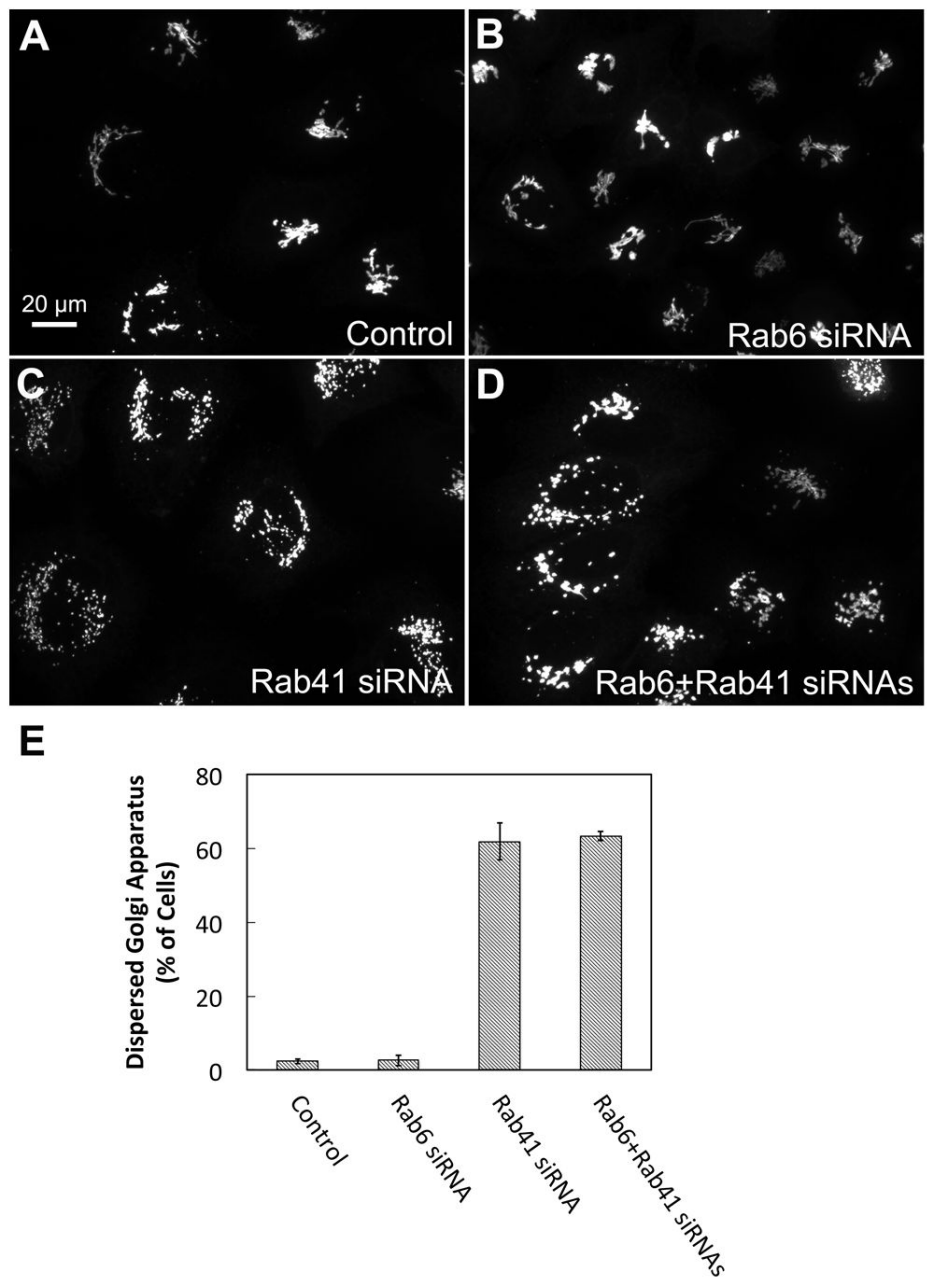
Co-depletion of Rab6 and Rab41 causes Golgi fragmentation, a result similar with Rab41 knockdown alone. HeLa cells stably expressing GalNAcT2-GFP were transfected with control, Rab6, Rab41 siRNA or both Rab6 and Rab41 siRNAs at an individual concentration of 200 nM. Cells were fixed 96 h post initial transfection. Rab6 knockdown alone had no effect on Golgi ribbon structure (B). However, when Rab6 and Rab41 were co-depleted, the Golgi apparatus was fragmented (D). This Golgi phenotype is similar with that of Rab41 knockdown alone (C). (E) Quantitative analysis of cells displayed fragmented Golgi. Error bars represent the mean ± SEM of three replicates. ~100 cells were assayed for each condition.

Finally, we investigated the effect of co-depletion of Rab41 and Rab6 on the Golgi ribbon organization. siRNA directed against nucleotide sequences common to Rab6a and a’ mRNA was used to knock down both. Rab6 knockdown alone produced little change in the Golgi ribbon structure (Figure 11B), while Rab41 knockdown alone, as expected, fragmented the Golgi apparatus to a clustered punctate Golgi distribution (Figure 11C). With co-depletion of Rab6 and Rab41, the Golgi apparatus was fragmented to an extent similar to the Rab41 knockdown alone (Figure 11D). Quantitatively, >60% of cells incubated with Rab41 directed siRNA alone or together with Rab6 directed siRNA had fragmented Golgi apparatus. Less than 5% incidence of dispersed Golgi apparatus was observed in control or Rab6-depleted cells (Figure 11E). In conclusion, the simplest interpretation of these results overall is that Rab41 and Rab6 affect Golgi organization in a parallel and independent manner. Therefore, together with the evidence that Rab6c regulates centrosome duplication and cell cycle progression rather than Golgi function [17], it can be concluded that the Rab VI subfamily, although related by homology and structure, shows considerable diversity in its function.

## Discussion

The previous studies of Pereira-Leal and Seabra [10] and Stein et al. [11] showed, based on either homology or protein structure, that Rab41 was a member of the same Rab subfamily as Rab6. Of the four closely related Rab6 isoforms, Rab6a/a’ is the most abundant Golgi-associated Rab proteins and has been studied most extensively (e.g., [15,18–20,22]). Rab6b functions in a similar manner in brain cells [16]; Rab6c is unique and regulates mitotic function rather than Golgi function [17]. Following from subfamily identification, our initial experimental goal was to determine if Rab41 had similar functional properties to Rab6a/a’. Surprisingly, our data showed that the functional role of Rab41 in Golgi organization and trafficking was in many ways the opposite of Rab6. The same conclusion followed from protein localization data.

The outcomes of both our knockdown and overexpression experiments indicate that Rab41 is actively involved in maintenance of Golgi ribbon structure. In knockdown experiments, depletion of Rab41 by siRab41(4) fragmented the Golgi apparatus. In cells overexpressing GDP-locked Rab41, but not wild type or GTP-locked Rab41, a dispersed Golgi ribbon was observed. At the cisternae and vesicle revealing resolution of electron microscopy, Rab41 depleted cells had comparatively short Golgi stacks. Each Golgi cisternal stack was relatively isolated and surrounded by an increased number of vesicles. These outcomes are strikingly different than the Golgi phenotype in Rab6-depleted cells, in which the Golgi ribbon is not disrupted [15] and the continuity of Golgi cisternae is increased, as is cisternal number per stack [23]. Consistent with contrasting phenotype, the localization of Rab41 and Rab6 in HeLa cells was found to be different. Rab6 is localized to the Golgi apparatus and, in particular, the trans-Golgi cisternae and TGN membranes [13–15]. Our results indicate that little, if any, Rab41 is associated with the Golgi apparatus. In brief, tagged, wild type and GTP-locked Rab41, but not GDP-locked Rab41, displayed occasional punctate localization that often concentrated in ruffled regions at the cell periphery. Significantly, we found that co-depletion of Rab6 and Rab41 did not suppress the Golgi fragmentation induced by Rab41 knockdown. Based on these results, we conclude that Rab41 and Rab6 act in parallel and likely independently to influence Golgi ribbon organization and underlying Golgi stack organization in ways that often appear opposing in function.

The fact that we found Rab41 was involved in supporting ER-to-Golgi trafficking in VSV-G transport experiments suggests a possible common theme between Rab41 function in Golgi ribbon phenotype and that of Rab1, Rab2, Rab18, and Rab43, all Rab proteins belonging to other Rab protein subfamilies. All these Rab proteins affect ER-to-Golgi trafficking. Depletion or inactivation of any of these Rab proteins affects Golgi ribbon organization with a typical phenotype being Golgi fragmentation to dispersal. Likely, continuous and rapid input of anterograde vesicles is needed to maintain Golgi organization [26–28]. Hence, the effect of Rab41 inactivation on Golgi organization could be an indirect effect. Our evidence that overexpression of wild type or GTP-locked Rab41 did not produce Golgi fragmentation is consistent with that interpretation. One effector candidate is RUSC2, which binds to Rab41 through its RUN motif [29] and has the potential to be an important regulatory factor for Rab41. The detailed identification and characterization of Rab41 effectors in future experiments will be an important step towards mechanistic understanding.

In conclusion, we found that Rab41 was actively required for the maintenance of the juxtanuclear Golgi ribbon in mammalian cells and normal rates of ER-to-Golgi trafficking. It acts in several ways in a manner that appears opposing to Rab6, a member of the same protein subfamily. By both siRNA depletion and overexpression of GDP-locked Rab41, the protein appears to be essential for cell growth and multiplication. In providing functional characterization of the role of Rab41, we have provided the first data on the last Rab protein of this small Rab protein branch. Of the five members, four, Rab6a/a’, Rab6b, and Rab41, have some role, be it direct or indirect, in Golgi ribbon organization and associated trafficking events. However, that role appears to be diverse (e.g., [15,16,18–20,23]). Rab6c has no role in membrane trafficking and the Golgi apparatus, but rather interacts with the centrosome [17]. Based on these results, neither the homology nor structure grouping appears to give a good indication of the actual functional role of these 5 proteins [10,11].

## Materials and Methods

### Cell culture

Wild type HeLa cells were grown in DMEM supplemented with 10% fetal bovine serum (FBS) in a humidified incubator at 37° C and 5% CO_2_. HeLa cells stably expressing tagged Golgi apparatus protein GalNAcT2-GFP were maintained in complete culture media in the presence of 0.45 mg/ml of Geneticin. All cell culture media, sera and associated reagents were obtained from either Invitrogen (Carlsbad, CA) or Sigma-Aldrich (St. Louis, MO).

### RNA interference

All small interfering RNAs (siRNAs) were synthesized by Dharmacon RNA Technologies (Lafayette, CO). The sense sequences of the four Rab41 siRNAs were

[siRab41(1)]: UUGCAGUGGUUGUCUAUGAUU;
[siRab41(2)]: CCUAAUUCCUAGCUACAUUUU;
[siRab41(3)]: GGAAUUGACUUCUUGUCUAUU;

[siRab41(4)]: GGAGACAGAUAAGUGGGUAUU starting at nucleotide positions 319, 285, 193 and 360 of the Rab41 coding sequence, respectively. Sequence of Rab6 siRNA (depletes both Rab6a and a’) has been published previously [15]. Control siRNA was siControl, non-targeting siRNA (UAGCGACUAAACACAUCAA). siRNA duplexes were transfected at a final concentration of 200 nM unless otherwise specified, using Oligofectamine (Invitrogen, Carlsbad, CA) according to the manufacturer’s protocol with minor modifications. In brief, ~70,000 HeLa cells stably expressing Golgi enzyme GalNAcT2-GFP were plated per 35 mm tissue culture dish. Cells were cultured overnight and then transfected with the corresponding siRNA in the absence of FBS for 4 h, 0 time, and 24 h later in order to achieve maximal knockdown penetrance [15,18]. Cells were fixed with formaldehyde 96 h after the first transfection cycle unless otherwise specified.

### Real-time PCR

HeLa cells were collected at 96 h post initial transfection. Total RNA was isolated from cells using RNeasy Protect Mini Kit (Qiagen, Gaithersburg, MD) according to the manufacturer’s instruction. 1 µg RNA was applied to synthesize cDNA by iScript^TM^ cDNA Synthesis Kit (Bio-Rad, Hercules, CA). The primers synthesized by Invitrogen (Carlsbad, CA) were (1) Rab41: 5’-AGACAAGTCACTGCAGAACAGGGT-3’ (forward primer) and 5’-AGTCCTTGTGGAAAGAAGGGCAGA-3’ (reverse primer); (2) GAPDH: 5’-GAGTCCACTGGCGTCTTCAC-3’ (forward primer) and 5’-TTCACACCCATGACGAACAT-3’ (reverse primer). Real-time PCR was performed on the Applied Biosystems Fast 7500HT real-time PCR system with a 15 µl reaction volume containing 1x of iQ SYBR Green Supermix (Bio-Rad, Hercules, CA), 100 ng of cDNA and 300 nM each primers. The standard cycling condition was 50° C for 2 min, 90° C for 10 min, followed by 40 cycles of 95° C for 15 s and 62° C for 1 min. The results were analyzed by SDS 2.3 relative quantification manager software. Relative quantification of mRNA expression was determined using comparative threshold cycle (*Ct*) method. All reactions were performed in triplicate and averaged.

### Plasmid construction

cDNA fragments expressing myc-tagged wild type, GTP-locked and GDP-locked Rab41 were synthesized and inserted into pUC57 vector by GeneScript (Piscataway, NJ), respectively. Two copies of BamHI sites at both 5’ end and 3’ end were also included in each sequence. pUC57 plasmid with insertion was digested by BamHI. The restriction fragment was purified and ligated into BamHI-digested pCMUIV, the mammalian expression vector used in all experiments. Inserts in all plasmids constructed were confirmed by sequencing.

### Microinjection of pCMUIV plasmids

An Eppendorf microinjection system was used as described previously [21,30]. HeLa cells stably expressing GalNAcT2-GFP were grown on glass coverslips for 2 days before microinjection. Purified pCMUIV plasmids were injected into the cell nuclei. During microinjection, cells were maintained at room temperature in CO_2_-independent medium (Invitrogen, Carlsbad, CA) supplemented with 2% FBS, and ~400 cells were injected per coverslip. DNA concentrations ranged from 25 to 200 ng/µl. Expression time was either 1 h or ranged from 8 h to 36 h. Cells were finally fixed and stained with anti-myc antibody as described previously [21,31].

### High-pressure freezing, freeze-substitution and electron microscopy

Cells were cultured on sapphire discs coated in a grid pattern with 20 nm thick carbon layer. Grid squares and numbers were used as guides to identify appropriate regions to later section. HeLa cells stably expressing GalNAcT2-GFP were transfected with either siRab41(4) or non-targeting siRNA duplexes as described above. Sapphire discs with siRab41(4) treated cells were imaged to find cells with fragmented Golgi apparatus. High-pressure freezing was performed essentially as described previously using a Leica EM PACT2 high-pressure freezing unit (Leica Microsystems) [32,33]. 2% Type IX ultra-low temperature gelling agarose (Sigma-Aldrich, St. Louis, MO) in PBS supplemented with 2% FBS was used as cryoprotectant. All solutions and sample holders (Swiss Precision Instruments) for high-pressure freezing were pre-warmed to 37° C. All manipulations were carried out on a heating block warmed to 37° C monitored with a dissecting microscope (Leica Microsystems). After high-pressure freezing, frozen cells were stored in liquid nitrogen.

Specimens were freeze-substituted with anhydrous acetone containing 2% OsO_4_/0.1% glutaraldehyde/1% H_2_O at -90° C for 16-22 h using a Leica AFS unit (Leica Microsystems). After specimens were warmed to 0°C over 2 days, they were immediately moved to the cold room (4° C). In the cold room, the specimens were incubated with acetone containing 1% tannic acid/1% H_2_O for 1 h, then replaced with acetone containing 1% OsO_4_/1% H_2_O and incubated for 1 h. Sapphire discs were rinsed repeatedly with acetone before and after each of incubation. After that, samples were warmed to room temperature, and then plastic embedded essentially as described previously [34].

For Rab41 depleted samples, Golgi apparatus fragmentation was verified by light microscopy before sectioning. Thin sections (50 nm) cut with a Leica UltraCut-UCT microtome were collected and post-stained with aqueous uranyl acetate and Reynold’s lead citrate (Electron microscopy Sciences) to enhance contrast. Images depicting representative Golgi regions in both Rab41 depleted and control cells were taken using a Tecnai F20 intermediate-voltage electron microscope operated at 80 keV (FEI Co.).

### VSV-G expression and chase

For testing the effect of Rab41 knockdown on VSV-G transport, wild type HeLa cells were treated with either siRab41(4) or non-targeting siRNA duplexes as described above. Typically after 96 h, cells were transfected with plasmid encoding tsO45 mutant of VSV-G-GFP using FuGENE HD Transfection reagent (Promega, Madison, WI) according to the manufacturer’s protocol. Cells were then incubated overnight at 39.5° C, non-permissive conditions for the transport of VSV-G from the ER to Golgi apparatus. After that, cells were shifted to 32° C, permissive conditions for tsO45-G transport and incubated for various chase in the presence of cycloheximide to prevent further protein synthesis. Cells were then fixed with formaldehyde and cell surface stained for VSV-G.

For testing effect of overexpression of GDP-locked Rab41 on VSV-G transport, a mixture of 100 ng/µl plasmid encoding myc-tagged GDP-locked Rab41 and 25 ng/µl plasmid encoding tsO45 mutant of VSV-G-GFP was injected into wild type HeLa cells. Cells injected only with 25 ng/µl plasmid encoding tsO45 mutant of VSV-G-GFP were used as control. Microinjection was performed as described above. Cells were then incubated at 39.5° C for 24 h, non-permissive conditions for the transport of VSV-G from the ER to Golgi apparatus. After that, cells were shifted to 32° C, permissive conditions for tsO45-G transport and incubated for 40 min in the presence of cycloheximide to prevent further protein synthesis. Cells were then fixed with formaldehyde and stained with anti-myc antibody to show the expression of GDP-locked Rab41.

### SLTB internalization

HeLa cells stably expressing GalNAcT2-GFP plated on coverslips were transfected with either siRab41(4) or non-targeting siRNA duplexes as described above. Typically after 96 h, these coverslips were transferred to ice-cold CO_2_-independent medium (Invitrogen, Carlsbad, CA) supplemented with 10% FBS. SLTB internalization was performed as described previously [35]. In brief, Cy3-SLTB was added at a final concentration of 70 µg/ml and incubated with cells at 4° C for 30 min to allow binding of SLTB to the cell surface. Cells were then washed with ice-cold CO_2_-independent media and transferred to 19.5° C CO_2_-independent media, and incubated at 19.5° C for 1 h in the presence of cycloheximide to allow accumulation of Cy3-SLTB in endosomes. After that, cells were washed with complete culture media and transferred to 37° C media and incubated for 20 min or 60 min in the presence of cycloheximide. Cells were fixed with formaldehyde at the end of the chase period.

### Fluorescence microscopy and image processing

For ER co-localization test of GTP-locked Rab41, HeLa cells stably expressing GalNAcT2-GFP were microinjected with plasmid encoding myc-tagged GTP-locked Rab41 at a concentration of 25 ng/µl. After 1-h expression, cells were fixed and double immunostained for myc-tagged Rab41 and Sec61p (ER marker). The injected cells were wide field imaged using a 63x/1.40 numerical aperture objective and a Zeiss 200M inverted microscope. All the images were then single-plane deconvolved and the extent of co-localization of GTP-locked Rab41 and Sec61p was quantified for 19 cells (using Huygens Pro software (SVI, Hilversum, the Netherlands).

For quantification of VSV-G distribution, cells expressing VSV-G-GFP were wide-field imaged using a 63x/1.40 numerical aperture objective and a Zeiss 200M inverted microscope. All these images were single-plane deconvolved. VSV-G distribution in Golgi apparatus or ER was determined by outlining the appropriate areas manually. The mean pixel intensity in the Golgi area and ER area was determined with iVision-MAC^TM^ software (BioVision Technologies, Exton, PA). To quantify cell surface VSV-G, cells expressing VSV-G-GFP were surface labeled for extracellular VSV-G epitope, imaged and then single-plane deconvolved. Cells were outlined manually. The mean pixel intensity was determined with iVision-MAC^TM^ software. All pixel intensities were corrected for background fluorescence as determined by outlining non-cellular areas within the images.

For quantification of the effect of siRNA treatment on cell growth, images were captured with a 10x phase contrast objective every 24 h. Six cell fields each were selected randomly. The number of cells per field was counted manually.

All other images were single plane projections of full cell depth, confocal image stacks produced by 63x/1.40 numerical aperture objective and a BD CARV II spinning disk confocal accessory mounted on a Zeiss 200M inverted microscope. Images were processed with iVision-MAC^TM^ software. For quantification of fragmented Golgi apparatus in knockdown experiments, several cell fields for each condition were selected randomly. The number of cells per field was counted manually. For quantification in microinjection experiments, the number of injected cells and injected cells displayed fragmented Golgi apparatus on each coverslip was counted manually. All scatter plots were prepared with KaleidaGraph 4.1 (Synergy software, Reading, PA).

## References

[1] Marra P, Salvatore L, Mironov A Jr, Di Campli A, Di Tullio G et al. (2007) The biogenesis of the Golgi ribbon: the roles of membrane input from the ER and of GM130. Mol Biol Cell 18: 1595-1608. doi:10.1091/mbc.E06-10-0886. PubMed: 17314401. 17314401 10.1091/mbc.E06-10-0886 PMC1855007

[2] Lowe M (2011) Structural organization of the Golgi apparatus. Curr Opin Cell Biol 23: 85-93. doi:10.1016/j.ceb.2010.10.004. PubMed: 21071196. 21071196 10.1016/j.ceb.2010.10.004

[3] Nilsson T, Au CE, Bergeron JJ (2009) Sorting out glycosylation enzymes in the Golgi apparatus. FEBS Lett 583: 3764-3769. doi:10.1016/j.febslet.2009.10.064. PubMed: 19878678. 19878678 10.1016/j.febslet.2009.10.064

[4] Surma MA, Klose C, Simons K (2012) Lipid-dependent protein sorting at the trans-Golgi network. Biochim Biophys Acta 1821: 1059-1067. doi:10.1016/j.bbalip.2011.12.008. PubMed: 22230596. 22230596 10.1016/j.bbalip.2011.12.008

[5] Lee MC, Miller EA, Goldberg J, Orci L, Schekman R (2004) Bi-directional protein transport between the ER and Golgi. Annu Rev Cell Dev Biol 20: 87-123. doi:10.1146/annurev.cellbio.20.010403.105307. PubMed: 15473836. 15473836 10.1146/annurev.cellbio.20.010403.105307

[6] Sandvig K, Spilsberg B, Lauvrak SU, Torgersen ML, Iversen TG et al. (2004) Pathways followed by protein toxins into cells. Int J Med Microbiol 293: 483-490. doi:10.1078/1438-4221-00294. PubMed: 15149022. 15149022 10.1078/1438-4221-00294

[7] Johannes L, Wunder C (2011) Retrograde transport: two (or more) roads diverged in an endosomal tree? Traffic 12: 956-962. doi:10.1111/j.1600-0854.2011.01200.x. PubMed: 21463456. 21463456 10.1111/j.1600-0854.2011.01200.x

[8] Stenmark H (2009) Rab GTPases as coordinators of vesicle traffic. Nat Rev Mol Cell Biol 10: 513-525. doi:10.1038/nrg2642. PubMed: 19603039. 19603039 10.1038/nrm2728

[9] Liu S, Storrie B (2012) Are Rab proteins the link between Golgi organization and membrane trafficking? Cell Mol Life Sci 69: 4093-4106. doi:10.1007/s00018-012-1021-6. PubMed: 22581368. 22581368 10.1007/s00018-012-1021-6 PMC4080914

[10] Pereira-Leal JB, Seabra MC (2001) Evolution of the Rab family of small GTP-binding proteins. J Mol Biol 313: 889-901. doi:10.1006/jmbi.2001.5072. PubMed: 11697911. 11697911 10.1006/jmbi.2001.5072

[11] Stein M, Pilli M, Bernauer S, Habermann BH, Zerial M et al. (2012) The interaction properties of the human Rab GTPase family--comparative analysis reveals determinants of molecular binding selectivity. PLOS ONE 7: e34870. doi:10.1371/journal.pone.0034870. PubMed: 22523562. 22523562 10.1371/journal.pone.0034870 PMC3327705

[12] Echard A, Opdam FJ, de Leeuw HJ, Jollivet F, Savelkoul P et al. (2000) Alternative splicing of the human Rab6A gene generates two close but functionally different isoforms. Mol Biol Cell 11: 3819-3833. doi:10.1091/mbc.11.11.3819. PubMed: 11071909. 11071909 10.1091/mbc.11.11.3819 PMC15039

[13] Antony C, Cibert C, Géraud G, Santa Maria A, Maro B et al. (1992) The small GTP-binding protein rab6p is distributed from medial Golgi to the trans-Golgi network as determined by a confocal microscopic approach. J Cell Sci 103 ( Pt 3): 785-796 PubMed: 1478971. 10.1242/jcs.103.3.785 1478971

[14] Goud B, Zahraoui A, Tavitian A, Saraste J (1990) Small GTP-binding protein associated with Golgi cisternae. Nature 345: 553-556. doi:10.1038/345553a0. PubMed: 2112230. 2112230 10.1038/345553a0

[15] Sun Y, Shestakova A, Hunt L, Sehgal S, Lupashin V et al. (2007) Rab6 regulates both ZW10/RINT-1 and conserved oligomeric Golgi complex-dependent Golgi trafficking and homeostasis. Mol Biol Cell 18: 4129-4142. doi:10.1091/mbc.E07-01-0080. PubMed: 17699596. 17699596 10.1091/mbc.E07-01-0080 PMC1995728

[16] Opdam FJ, Echard A, Croes HJ, van den Hurk JA, van de Vorstenbosch RA et al. (2000) The small GTPase Rab6B, a novel Rab6 subfamily member, is cell-type specifically expressed and localised to the Golgi apparatus. J Cell Sci 113 ( Pt 15): 2725-2735 PubMed: 10893188. 10.1242/jcs.113.15.2725 10893188

[17] Young J, Ménétrey J, Goud B (2010) RAB6C is a retrogene that encodes a centrosomal protein involved in cell cycle progression. J Mol Biol 397: 69-88. doi:10.1016/j.jmb.2010.01.009. PubMed: 20064528. 20064528 10.1016/j.jmb.2010.01.009

[18] Young J, Stauber T, del Nery E, Vernos I, Pepperkok R et al. (2005) Regulation of microtubule-dependent recycling at the trans-Golgi network by Rab6A and Rab6A'. Mol Biol Cell 16: 162-177. PubMed: 15483056. 15483056 10.1091/mbc.E04-03-0260 PMC539161

[19] Miserey-Lenkei S, Chalancon G, Bardin S, Formstecher E, Goud B et al. (2010) Rab and actomyosin-dependent fission of transport vesicles at the Golgi complex. Nat Cell Biol 12: 645-654. doi:10.1038/ncb2067. PubMed: 20562865. 20562865 10.1038/ncb2067

[20] Grigoriev I, Yu KL, Martinez-Sanchez E, Serra-Marques A, Smal I et al. (2011) Rab6, Rab8, and MICAL3 cooperate in controlling docking and fusion of exocytotic carriers. Curr Biol 21: 967-974. doi:10.1016/j.cub.2011.04.030. PubMed: 21596566. 21596566 10.1016/j.cub.2011.04.030

[21] Jiang S, Storrie B (2005) Cisternal rab proteins regulate Golgi apparatus redistribution in response to hypotonic stress. Mol Biol Cell 16: 2586-2596. doi:10.1091/mbc.E04-10-0861. PubMed: 15758030. 15758030 10.1091/mbc.E04-10-0861 PMC1087260

[22] Micaroni M, Stanley AC, Khromykh T, Venturato J, Wong CX et al. (2013) Rab6a/a’ are important Golgi regulators of pro-inflammatory TNF secretion in macrophages. PLOS ONE 8: e57034. doi:10.1371/journal.pone.0057034. PubMed: 23437303. 23437303 10.1371/journal.pone.0057034 PMC3578815

[23] Storrie B, Micaroni M, Morgan GP, Jones N, Kamykowski JA et al. (2012) Electron tomography reveals Rab6 is essential to the trafficking of trans-Golgi clathrin and COPI-coated vesicles and the maintenance of Golgi cisternal number. Traffic 13: 727-744. doi:10.1111/j.1600-0854.2012.01343.x. PubMed: 22335553. 22335553 10.1111/j.1600-0854.2012.01343.x PMC3324626

[24] Miserey-Lenkei S, Couëdel-Courteille A, Del Nery E, Bardin S, Piel M et al. (2006) A role for the Rab6A’ GTPase in the inactivation of the Mad2-spindle checkpoint. EMBO J 25: 278-289. doi:10.1038/sj.emboj.7600929. PubMed: 16395330. 16395330 10.1038/sj.emboj.7600929 PMC1383512

[25] Martinez O, Schmidt A, Salaméro J, Hoflack B, Roa M et al. (1994) The small GTP-binding protein rab6 functions in intra-Golgi transport. J Cell Biol 127: 1575-1588. doi:10.1083/jcb.127.6.1575. PubMed: 7798313. 7798313 10.1083/jcb.127.6.1575 PMC2120294

[26] Dejgaard SY, Murshid A, Erman A, Kizilay O, Verbich D et al. (2008) Rab18 and Rab43 have key roles in ER-Golgi trafficking. J Cell Sci 121: 2768-2781. doi:10.1242/jcs.021808. PubMed: 18664496. 18664496 10.1242/jcs.021808

[27] Haas AK, Yoshimura S, Stephens DJ, Preisinger C, Fuchs E et al. (2007) Analysis of GTPase-activating proteins: Rab1 and Rab43 are key Rabs required to maintain a functional Golgi complex in human cells. J Cell Sci 120: 2997-3010. doi:10.1242/jcs.014225. PubMed: 17684057. 17684057 10.1242/jcs.014225

[28] Tisdale EJ, Bourne JR, Khosravi-Far R, Der CJ, Balch WE (1992) GTP-binding mutants of rab1 and rab2 are potent inhibitors of vesicular transport from the endoplasmic reticulum to the Golgi complex. J Cell Biol 119: 749-761. doi:10.1083/jcb.119.4.749. PubMed: 1429835. 1429835 10.1083/jcb.119.4.749 PMC2289685

[29] Fukuda M, Kobayashi H, Ishibashi K, Ohbayashi N (2011) Genome-wide investigation of the Rab binding activity of RUN domains: development of a novel tool that specifically traps GTP-Rab35. Cell Struct Funct 36: 155-170. doi:10.1247/csf.11001. PubMed: 21737958. 21737958 10.1247/csf.11001

[30] Storrie B (2005) Microinjection as a tool to explore small GTPase function. Methods Enzymol 404: 26-42. doi:10.1016/S0076-6879(05)04004-8. PubMed: 16413255. 16413255 10.1016/S0076-6879(05)04004-8

[31] Shestakova A, Zolov S, Lupashin V (2006) COG complex-mediated recycling of Golgi glycosyltransferases is essential for normal protein glycosylation. Traffic 7: 191-204. doi:10.1111/j.1600-0854.2005.00376.x. PubMed: 16420527. 16420527 10.1111/j.1600-0854.2005.00376.x

[32] McDonald K, Schwarz H, Müller-Reichert T, Webb R, Buser C et al. (2010) "Tips and tricks" for high-pressure freezing of model systems. Methods Cell Biol 96: 671-693. doi:10.1016/S0091-679X(10)96028-7. PubMed: 20869543. 20869543 10.1016/S0091-679X(10)96028-7

[33] Verkade P (2008) Moving EM: the Rapid Transfer System as a new tool for correlative light and electron microscopy and high throughput for high-pressure freezing. J Microsc 230: 317-328. doi:10.1111/j.1365-2818.2008.01989.x. PubMed: 18445162. 18445162 10.1111/j.1365-2818.2008.01989.x

[34] Marsh BJ, Mastronarde DN, Buttle KF, Howell KE, McIntosh JR (2001) Organellar relationships in the Golgi region of the pancreatic beta cell line, HIT-T15, visualized by high resolution electron tomography. Proc Natl Acad Sci U S A 98: 2399-2406. doi:10.1073/pnas.051631998. PubMed: 11226251. 11226251 10.1073/pnas.051631998 PMC30150

[35] Starr T, Sun Y, Wilkins N, Storrie B (2010) Rab33b and Rab6 are functionally overlapping regulators of Golgi homeostasis and trafficking. Traffic 11: 626-636. doi:10.1111/j.1600-0854.2010.01051.x. PubMed: 20163571. 20163571 10.1111/j.1600-0854.2010.01051.x

